# Development of a Curcumin-Loaded Lecithin/Chitosan Nanoparticle Utilizing a Box-Behnken Design of Experiment: Formulation Design and Influence of Process Parameters

**DOI:** 10.3390/polym14183758

**Published:** 2022-09-08

**Authors:** Ismail A. Walbi, Mohammad Zaki Ahmad, Javed Ahmad, Mohammed S. Algahtani, Amer S. Alali, Samar A. Alsudir, Alhassan H. Aodah, Hassan A. Albarqi

**Affiliations:** 1 Department of Clinical Pharmacy, College of Pharmacy, Najran University, Najran 11001, Saudi Arabia; 2 Department of Pharmaceutics, College of Pharmacy, Najran University, Najran 11001, Saudi Arabia; 3 Department of Pharmaceutics, College of Pharmacy, Prince Sattam Bin Abdulaziz University, Al-Kkharj 11942, Saudi Arabia; 4 National Center of Biotechnology, Life Science and Environment Research Institute, King Abdulaziz City for Science and Technology (KACST), Riyadh 11442, Saudi Arabia

**Keywords:** curcumin, chitosan, lecithin, nanoparticles, stirring speed, 3-factorial Box-Behnken statistical design

## Abstract

Curcumin (CUR) has impressive pharmacologic properties, including cardioprotective, neuroprotective, antimicrobial, and anticancer activity. However, the pharmaceutical application of CUR is limited due to its poor aqueous solubility and low bioavailability. The development of novel formulations has attracted considerable attention to the idea of applying nanobiotechnology to improve the therapeutic efficacy of these challenging compounds. In this study, CUR-loaded lecithin–chitosan nanoparticles (CUR/LCSNPs) were developed and optimized by the concentration of chitosan, lecithin, and stirring speed by a 3-factorial Box-Behnken statistical design, resulting in an optimal concentration of chitosan (*A*) and lecithin (*B*) with a 1200 rpm stirring speed (*C*), with applied constraints of minimal average particle size (Y_1_), optimal zeta potential (Y_2_), and maximum entrapment efficiency (%EE) (Y_3_). The mean particle size of the checkpoint formulation ranged from 136.44 ± 1.74 nm to 267.94 ± 3.72, with a zeta potential of 18.5 ± 1.39 mV to 36.8 ± 3.24 mV and %EE of 69.84 ± 1.51% to 78.50 ± 2.11%. The mean particle size, zeta potential, %EE, and % cumulative drug release from the optimized formulation were 138.43 ± 2.09 nm, +18.98 ± 0.72 mV, 77.39 ± 1.70%, and 86.18 ± 1.5%, respectively. In vitro drug release followed the Korsmeyer–Peppas model with Fickian diffusion (*n* < 0.45). The optimized technique has proven successful, resulting in a nanoformulation that can be used for the high loading and controlled release of lipophilic drugs.

## 1. Introduction

Curcumin (CUR) has been used for centuries as a conventional remedy both in ancient Indian and Chinese medicine [1]. It is derived from the rhizome of the perennial herb Curcuma longa (Zingiberaceae family) and is widely called turmeric [2]. CUR has shown remarkable pharmacologic efficacy as cardioprotective, neuroprotective, anti-microbial, and anticancer [1,2,3]. Furthermore, various clinical and preclinical studies demonstrated that CUR is considerably non-toxic at higher doses [4,5]. The fact that CUR exhibits such a wide range of safety and pharmacological efficacy increase its utility as a therapeutic agent for the prevention and treatment of a wide range of disease [6,7]. Despite CUR’s potential therapeutic efficacy, the limited bioavailability and the high variability in pharmacokinetics behavior limit its clinical application [6,7]. The bioavailability of CUR is limited by its poor aqueous solubility, inadequate tissue absorption, rapid systemic elimination, fast metabolism, and degradation at basic pH [1,2,6]. Furthermore, the lipophilic nature of CUR makes it prone to reticuloendothelial system uptake and decreases the therapeutic concentration at the desired site [8].

Various nanosystems have been utilized to increase the aqueous solubility and bioavailability of CUR by encapsulating it in a liposome, polymeric nanoparticle, lipid-based nanoparticle, biodegradable microsphere, nanoemulsion, nanoemulgel, etc. [9,10,11,12,13]. Nanoparticle (NP)-based drug delivery strategies have the potential to enhance the drug delivery of lipophilic therapeutics such as CUR through colloidal dispersion in an aqueous media, thereby avoiding the drawbacks associated with low aqueous solubility, and offer enhancement in the efficacy of loaded therapeutics [8,14]. Developing biodegradable novel drug delivery systems (NDDS) to increase the bioavailability of therapeutic agents have been the primary focus of research over the last few decades [8,15]. To meet the aforementioned requirements, drug delivery systems involving polymeric nanoparticles are emerging as promising alternatives. A controlled delivery system based on chitosan (CS) nanoparticles has been demonstrated to be effective for delivering various pharmacologically active compounds, including anticancer agents, analgesics, and peptides [16,17,18,19,20]. In the presence of various functional groups (like amino and hydroxyl groups), CS can form NPs by cross-linking with polymers with a polyanionic nature [15]. Recent studies have reported that negatively charged lecithin improves the drug delivery of poorly soluble compounds by interacting with positively charged CS. [21,22,23]. Alkholief developed CS and lecithin (L)-based NPs (LCSNP) for the simultaneous encapsulation and delivery of doxorubicin and piperine against doxorubicin-resistant cancer cells [21]. Furthermore, Lopes Rocha et al. developed LCS-based NPs encapsulated with melatonin (ML) for wound healing in diabetic rats [22]. Similarly, Murthy et al. demonstrated the formulation design of LCSNPs utilized to enhance the oral bioavailability of raloxifene hydrochloride [23]. In another study, Panda et al. reported the encapsulation of berberine inside LCSNP and evaluated wound-healing activity for improved therapeutic efficacy in a diabetic animal model [24]. A thymoquinone (TMQ)-loaded CS–lecithin delivery system for effective wound healing was reported by Negi et al. [25]. A TMQ-loaded lecithin–CS delivery system demonstrated the significantly enhanced wound-healing efficacy in comparison to the control [25]. TMQ-loaded LCSNPs induce earlier collagen deposition and wound contraction in comparison to TMQ alone [25]. In another study, Perez-Ruiz et al. demonstrated the significantly anticancer activity of (−)-epicatechin (EC)-loaded LCSNPs against different cancer cell lines (such as MDA-MB-231, MCF-7, SK-Br3, and MDA-MB-436) [26]. Ilk et al. investigated the formulation and evaluation of kaempferol-loaded lecithin–CS NPs for antifungal activity [27]. The developed formulation exhibited a significant inhibition efficacy (67%) against Fusarium oxysporum. All of the previous investigations discussed above provide proof-of-concept for the LCSNPs as promising nanocarriers to improve the biopharmaceutical performance of poorly soluble therapeutics.

In this study, we aimed to develop a CUR-loaded lecithin–CS-based self-assembled NPs drug delivery system. CUR-loaded LCSNPs (CUR/LCSNPs) were optimized through a quality-by-design (QbD) approach to understanding the impact of formulation-related factors and their interaction with the desirable attributes of the developed formulation system through a desired set of experiments. A Box-Behnken experimental design was employed in the current investigation as a statistical tool to optimize the concentration of formulation variables/process parameters and to investigate the combined influence of independent variables (concentration of nanoparticles components [chitosan, and lecithin], as well as the stirring speed used in its preparation) on dependent responses (particle size, surface charge, and entrapment efficiency). It helps to optimize the CUR-containing nanoparticulate system of specific dimensions as per the suitability requirement in a particular disease and/or its drug delivery prospects through a particular route of drug administration, which ultimately leads to the enhancement of the therapeutic efficacy of CUR at the target site.

## 2. Materials and Methods

### 2.1. Materials

Curcumin (CUR), low molecular chitosan (CS) (with viscosity average molecular weight of 50,000–190,000 Da, 75–85% deacetylated), isopropyl myristate (IPM), L-α-lecithin (L), and 95% *v*/*v* ethanol (ETOH) were obtained from Sigma Aldrich, (Taufkirchen, Germany). A 0.45 µm membrane filter was purchased from MF-Millipore^®^, Merck KGaA, (Darmstadt, Germany). Purified water was obtained from Milli-Q^®^ Type 1 Ultrapure Water Systems (Burlington, MA, USA).

### 2.2. Preparation of Curcumin Loaded Lecithin-Chitosan Nanoparticles

CUR/LCSNPs were prepared according to a method described by Sonvico et al. (2006) with some modifications [28]. Briefly, 10 mL of lecithin solutions of different concentrations (2.5%, 3.75%, and 5% *w*/*v*) were prepared by dissolving an appropriate quantity (250, 375, and 500 mg) of lecithin in 95% ETOH (containing 0.25 mL of IPM). Then, each solution of L was treated with 10 mg of CUR to yield a 0.1% *w*/*v* CUR solution. Similarly, an aqueous solution of CS (1% *w*/*v*) was prepared by dissolving 1 g of CS in 100 mL of an acetic acid solution (1%) with stirring overnight [23,24]. The chitosan solution was filtered [23]. An appropriate volume (0.5 mL, 1.25 mL, 2 mL) of CS (1% *w*/*v*) was further diluted with a (1% *v*/*v*) acetic acid solution to obtain a final volume of 23 mL [23,24]. Then, 2 mL of L-ethanolic CUR solution was injected using a 20-gauge needle into the 23 mL of diluted CS solution in 1% acetic acid under magnetic stirring for 60 min to formulate CUR/LCSNPs [21,28]. The CUR/LCSNPs suspension was then filtered under a vacuum. The filtrate was collected and centrifuged at 20,000 rpm for 30 min at 4 °C. The sediment was re-suspended with an appropriate volume of purified water.

### 2.3. Optimization of CUR/LCSNPs

In this optimization process, a 3-factorial Box-Behnken statistical design was applied to optimize CUR/LCSNPs through Design Expert^®^ software (version 13.0.5.0. Stat-Ease., Minneapolis, MN, USA). In this study, 3 factors and 3 levels of each variable (−1, 0, and +1) were applied to investigate the impact of independent variables (amount/concentration of chitosan, lecithin, and stirring speed during the preparation of CUR/LCSNPs) on dependent variables (particle size, zeta potential, and entrapment efficiency of CUR/LCSNPs) (Table 1). 

The amount of chitosan (*A*), lecithin (*B*), and the stirring speed (*C*) during the preparation were selected as independent variables, considering their overall impact, and the particle size (Y_1_), zeta potential (Y_2_), and entrapment efficiency (Y_3_) were selected as dependent variables. Seventeen formulations of different combinations, including five center points, were prepared according to the Box-Behnken design suggestions and are shown in Table 2. The observed response in the form of particle size, zeta potential, and %EE of all seventeen formulations of CUR/LCSNPs was recorded. All experiments were performed in triplicate, the results of which are presented as ±SD.

#### 2.3.1. Particle Size and Zeta Potential Measurements

The particle size and polydispersity index (PDI) of CUR/LCSNPs were measured using dynamic light scattering (DLS) techniques with the Nano ZS90 zeta sizer (Malvern Instruments, Malvern, UK). Purified water was used to dilute the samples (50 times) for analysis (Milli-Q^®^ Type 1 Ultrapure Water Systems) [29,30]. For the determination of zeta potential, the sample was transferred into disposable folded capillary cells (DTS1070), and the zeta potential was determined using the same instruments [31]. 

#### 2.3.2. Determination of Encapsulation Efficiency 

The encapsulation efficiency (%EE) was calculated using the following Equation (1), as previously reported in [21,22,24,27]. Briefly, the obtained supernatant from the prepared samples (as described in Section 2.2) was diluted and analyzed for free CUR concentration through a UV-visible spectrophotometer at λmax 425 nm [30].

(1) $$\%EE=\left(\frac{Initial\;amount\;of\;CUR\;\left(µg\right)-Free\;CUR\;in\;supernatant}{initial\;amount\;of\;CUR\;\left(µg\right)}\right)\times 100\;\;$$

ANOVA was used to statistically validate the polynomial equations created using the Box-Behnken design. Statistically, the significance of coefficient and r^2^ values for all the responses were evaluated by fitting them to linear, second-order (2FI), and quadratic models [32]. Different approaches were explored to determine the components of optimized NPs. The optimized CUR/LCSNPs were selected and characterized for the selected response. Prediction error (%) was calculated by comparing the actual response data and the predicted data [32].

#### 2.3.3. Scanning Electron Microscopy (SEM)

SEM characterization was performed using a JEOL SEM (JSM-IT500HR), ASIA PTE. Ltd., (Singapore city, Singapore). In preparation for imaging, samples were centrifuged at 15,000 rpm for 2 h, and then a drop of each sample’s supernatant was deposited on a SEM stub and left to dry before platinum coating (2 nm thickness) using the Auto Fine Coater (JEC-3000FC), JEOL, ASIA PTE. Ltd., Singapore. 

#### 2.3.4. X-ray Diffraction (XRD) Analysis

A Rigaku miniflex 300/600 (Tokyo, Japan), equipped with a 40 kV radiation source and a 15 mA current source, was used to investigate the different physical forms of curcumin, lecithin, chitosan, LCSNPs, and CUR/LCSNPs. The data recording was taken between 2θ ranging from 0° to 60° at a scan speed of 10°/min.

### 2.4. In Vitro Drug Release and Kinetics

The in vitro release profile of CUR from the optimized CUR/LCSNPs and an aqueous suspension of CUR were studied by the dialysis bag method [30,31]. A total of 2.0 mL of each CUR/LCSNP and an aqueous suspension of CUR were placed in the dialysis tube of molecular weight cutoff 12–14 kD (Spectra^®^ dialysis tubing, New Brunswick, NJ USA). Both ends of the dialysis tube were tightly sealed and then placed in the 1000 mL capacity beaker containing 500 mL of release media (phosphate buffer pH 7.4 with 5% ethyl alcohol as co-solubilizer) [30]. The whole system was maintained at 37 °C under constant stirring (100 rpm) with magnetic beads. Aliquots of 5 mL samples were withdrawn at the predetermined time points of 0, 0.5, 1, 2, 4, 6, 8, 10, 12, and 24 h and was replaced with fresh media. Withdrawn samples were suitably diluted, and the concentration of CUR in each sample was determined by UV–visible spectroscopy at λmax 425 nm [30]. The obtained release data of CUR from the optimized formulation systems were fitted to several kinetic models, including the zero-order, first-order, Higuchi, Korsmeyer–Peppas, and Hixon–Crowell models. The best fit model was identified based on the R^2^ value (approaching 1) [30]. 

### 2.5. Physical Stability Study

The physical stability of the optimized CUR/LCSNPs suspension was determined by the previously described method with a slight modification [22]. Briefly, a volume of 2 mL of CUR/LCSNPs was placed into screw-capped glass vials and stored at 30 ± 1 °C, away from direct sunlight, and the particle size, zeta potential, PDI, and %drug content during the storage conditions were monitored for 0, 7, 15, and 45 days.

## 3. Results and Discussion

### 3.1. Optimization of CUR/LCSNPs Utilizing a Box-Behnken Statistical Design

A 3-factor 3-level Box-Behnken design was used in this study to investigate the correlations between the dependent and independent factors of CUR/LCSNPs. The composition characteristics for the independent variables are shown in Table 2. 

The polynomial equation generated for this investigation is represented as follows

Y = α_0_ + α_1_*A* + α_2_*B* + α_3_*C* + α_12_*AB* + α_13_
*AC* + α_23_*BC* + α_11_*A*^2^ + α_22_*B*^2^ + α_33_*C*^2^ (2)

where Y = dependent variables; α_0_ is the intercept; α_1_, α_2_, and α_3_ represent the regression coefficient. *A*, *B*, and *C* are independent variables. Terms (*AB*, *AC*, and *BC*) and (*A*^2^, *B*^2^, and *C*^2^) represent the interaction and quadratic effect, respectively [32,33]. The quadratic model was the best-fitted model for all three dependent responses, as shown in Table 3. 

#### 3.1.1. Fitting of the Data on the Model Suggested by a Box-Behnken Statistical Design

The regression equation in terms of the coded factor was used to predict the effect of the interaction of independent factors (amount/concentration of chitosan, lecithin, and stirring speed during the preparation of CUR/LCSNPs) on responses. As indicated in Table 4, according to the ANOVA for the quadratic model, the model F-value of 26.81 for response (Y_1_) implies the model is significant. The probability of a larger F-value occurring due to noise is only 0.01. For the response (Y_2_), the F-value of 73.82 indicates that the model is significant. The probability of a larger F-value occurring due to noise is only 0.01.

Similarly, for the drug %EE, (Y_3_), the model F-value of 20.64 implies the model is significant. The probability of a larger F-value occurring due to noise is only 0.03. The *p*-value less than 0.05 indicates that the model factors are significant. The significant model factors were *A*, *C*, *AB*, *BC*, and *C*^2^ for (Y_1_); *A*, *B*, *BC* and *A*^2^ for (Y_2_); and *A*, *C*, *AB*, *B*^2^, and *C*^2^ for (Y_3_). The lack of fit F values such as 1.468 for (Y_1_), 0.7163 for (Y_2_), and 3.37 for (Y_3_) indicates that the results were not significant in comparison to the pure error. Furthermore, there is a 72.17% for (Y_1_), 59.18% for (Y_2_), and 13.58% (Y_3_) chance that noise could cause a lack of a larger F-value. 

#### 3.1.2. Interaction Effect of Independent Variables in the Responses 

In our study, we observed that lecithin and CS total amounts influence the mean particle size of the obtained CUR/LCSNPs. CS was found to have the greatest impact on the mean particle size compared to other independent factors (such as the amount of lecithin in the CUR/LCSNPs composition and the stirring speed applied during its preparation). The mathematical relationship obtained utilizing a Box-Behnken statistical design suggested a quadratic model with the following polynomial equation for mean particle size.

Y_1_ = 184.79 + 41.02*A* + 3.09*B* − 8.30*C* + 4.24*AB* − 10.25 *AC* + 0.169*BC* + 6.33*A*^2^ − 8.04*B*^2^ + 9.66*C*^2^ (3)

The interaction effect of *A* and *B* for the CUR/LCSNPs was found to have a positive impact on the response Y_1_, while *C* has a negative impact on Y_1_. The combined effect of *A* and *B* and *B* and *C* has a positive impact on Y_1_ (the impact effect of combined *A* and *B* is approximately 25 times higher than the effect of combined *B* and *C*), while the combined effect of *A* and *C* has a negative impact on Y_1_. The mathematical relationship obtained utilizing a Box-Behnken statistical design suggested a quadratic model with the following polynomial equations for mean particle size (Y_2_) and %EE (Y_3_).

Y_2_ = 28.93 + 7.36*A* − 2.10*B* − 0.0125*C* − 0.06*AB* + 0.075*AC* + 0.20*BC* − 2.10*A*^2^ + 0.22*B*^2^ + 0.345*C*^2^ (4)

Y_3_ = 71.07 + 2.57*A* + 0.51*B* + 1.65*C* − 3.06*AB* − 0.90*AC* + 0.722*BC* + 0.956*A*^2^ + 1.76*B*^2^ + 3.52*C*^2^ (5)

It was observed that the effect of *A* was found to have a positive impact on responses Y_2_, while *B* and *C* have a negative impact. For response Y_3_, the influence of *A*, *B*, and *C* has a positive impact on Y_3_. However, Y_3_ is negatively impacted by the interaction of *AB* and *AC*, while Y_3_ is positively impacted by *BC*. 

The mean particle size of the checkpoint formulation ranged from 136.44 ± 1.70 nm to 267.94 ± 3.72 nm. The amount of CS and lecithin in the different formulations (Table 2) has an influence on the mean particle size. The particle size of CUR/LCSNPs was 136.44 ± 1.70 nm in (F9) consisting of 5 mg of CS, while it was 267.94 ± 3.70 nm in (F17) consisting of 20 mg of CS. At the mean concentration (12.5 mg) of CS, the mean particle size was observed to be 189.40 ± 1.02 nm (F7) Table 2. The particle size was observed to be 162.43 ± 2.50 nm in (F11) consisting of 50 mg of lecithin, while it was 218.63 ± 2.70 nm in (F4) consisting of 75 mg of lecithin (Table 2). 

The stirring speed also influenced the mean particle size across the different formulations (Table 2). It was 267.94 ± 3.70 nm (F17) when the stirring speed was 900 rpm, while it became 228.63 ± 2.70 nm (F4) at 1500 rpm. The effect of the three independent factors on the mean particle size of CUR/LCSNPs was observed to be in the following order: amount of CS > amount of lecithin > stirring speed. 

**Figure 1:**
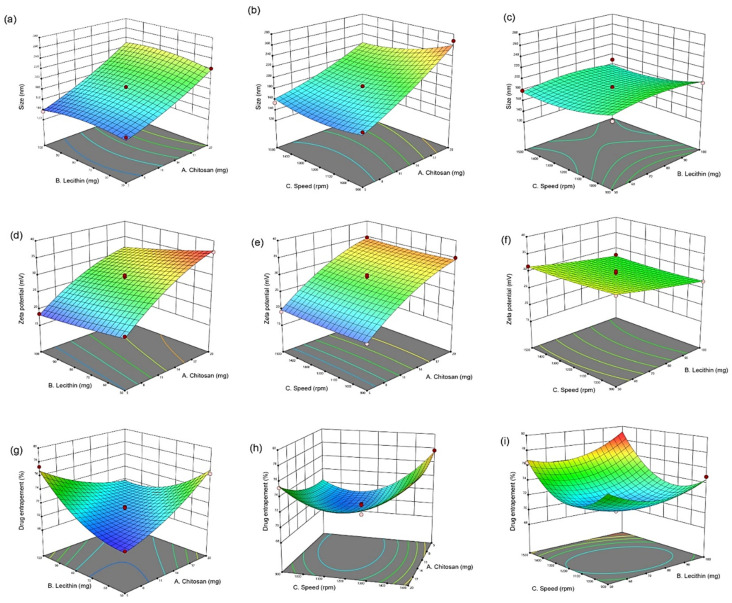
3D-response surface plots for the graphical optimization of CUR/LCSNPs, illustrating the interaction effect of (**a**) lecithin and chitosan on mean particle size. (**b**) Speed and chitosan on mean particle size. (**c**) Speed and lecithin on mean particle size. (**d**) Lecithin and chitosan on ZP. (**e**) Speed and chitosan on ZP. (**f**) Speed and lecithin on ZP. (**g**) Lecithin and chitosan on EE(%). (**h**) Speed and chitosan on EE (%). (**i**) Speed and lecithin on EE (%).

The effect of independent variables on the mean particle size of CUR/LCSNPs is illustrated in Figure 1a–c as 3D-response surface plots. The ANOVA test revealed a statistically significant relationship between the mean particle size (Y_1_) and independent variables at 95% CI (*p* < 0.05) for *A*, *B*, and *C*, as shown in Table 4. Furthermore, R^2^ fit statistics showed 99.84% of the mean particle size variability demonstrated by the fitted model.

The zeta potential value of the prepared CUR/LCSNPs was found to be in the range of +18.5 ± 1.40 to 36.8 ± 3.20 mV. The CS in the composition of CUR/LCSNPs is responsible for inducing a positive charge on the surface of CUR/LCSNPs. The increase in the positive value of zeta potential was observed with the increase in the CS concentration in all checkpoint formulations (F1–F17). There was no noticeable influence of lecithin on the inducing of the negative charge on the surface of CUR/LCSNPs. The effect of independent variables (amount/concentration of chitosan, lecithin, and stirring speed during the preparation of CUR/LCSNPs) on the zeta potential of CUR/LCSNPs is illustrated in Figure 1d–f as 3D-response surface plots. The ANOVA test revealed a statistically significant relationship between zeta potential (Y_2_) and independent variables at the 95% CI (*p* < 0.05) for *A*, *B*, and *C*, as shown in Table 4. Furthermore, R^2^ fit statistics revealed that 98.96% of the zeta potential variability demonstrated the fitted model. 

The quantity of CS and the stirring speed (rpm) applied during the preparation of CUR/LCSNPs were the main factors that influenced the %EE of CUR in developed CUR/LCSNPs formulations. The %EE of all checkpoint formulations suggested by the Box-Behnken statistical design varies from 69.84 ± 1.50% to 78.50 ± 2.10%. It was observed that the %EE of the prepared CUR/LCSNPs (F3) consisting of 5 mg CS was found to be 69.84 ± 1.50% while it was 76.33 ± 2.10% at (F6) consisting of 20 mg of CS. In the case of stirring speed, the %EE of prepared CUR/LCSNPs was found to be 71.46 ± 2.30% at (F11) when the stirring speed was 900 rpm, while it was 77.69 ± 2.70% at (F10) when the stirring speed was 1500 rpm. The influence of three designated independent factors on the %EE of CUR/LCSNPs was observed to be in the following order: quantity of CS > stirring speed (rpm) > quantity of lecithin. The independent variables’ effect on EE% of CUR/LCSNPs is illustrated in Figure 1g–i as 3D-response surface plots. The ANOVA test revealed a statistically significant relationship between %EE (Y_3_) and independent variables at the 95% CI (*p* < 0.05) for *A*, *B*, and *C*, as shown in Table 4. Furthermore, R^2^ fit statistics revealed that 98.37% of the %EE variability was demonstrated by the fitted model.

#### 3.1.3. Applied Model Validation

**Figure 2:**
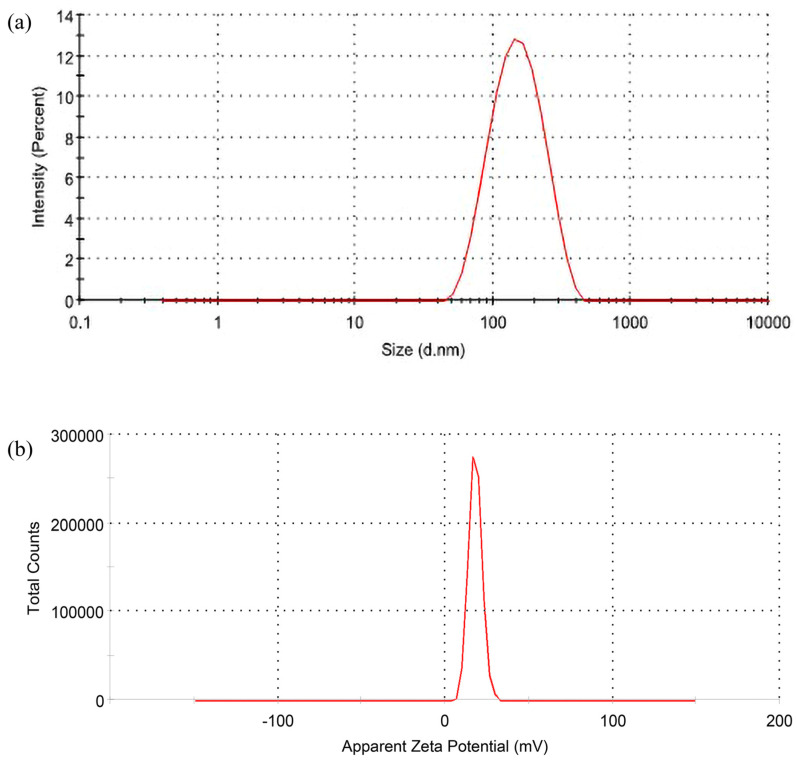
(**a**) Particle size distribution and (**b**) the zeta-potential of optimized CUR/LCSNPs.

The determination of the interaction effect of independent variables on dependent variables was desirable (Figure 1). The 3D plot displays the impact of two factors on a specific response, and the third factor remains constant. The optimized CUR/LCSNPs were selected based on the point-prediction methodology, i.e., the selection was based on attaining the minimum mean particle size and maximum entrapment efficiency (%EE) with optimal zeta potential [34]. A Box-Behnken statistical design model yielded the desirable values for mean particle size, zeta potential, and %EE CUR/LCSNPs of 141.40 nm, +18.19 mV, and 76.78%, respectively. Therefore, considering the suggested compositions of CUR/LCSNPs for the desirable predicted values of dependent variables, a desirable CUR/LCSNPs was prepared and evaluated to know the desirable value of the dependent variables of CUR/LCSNPs. It was found that desirable CUR/LCSNPs have a mean particle size of 138.4 ± 2.10 nm, a zeta potential of +18.98 ± 0.72 mV (Figure 2 a,b), and %EE of 77.40 ± 1.70. The experimental/observed values obtained for dependent variables of CUR/LCSNPs were found to be near the predicted values. The percentage prediction error was calculated and found to be within the permissible limit (as shown in Table 5).

**Figure 3:**
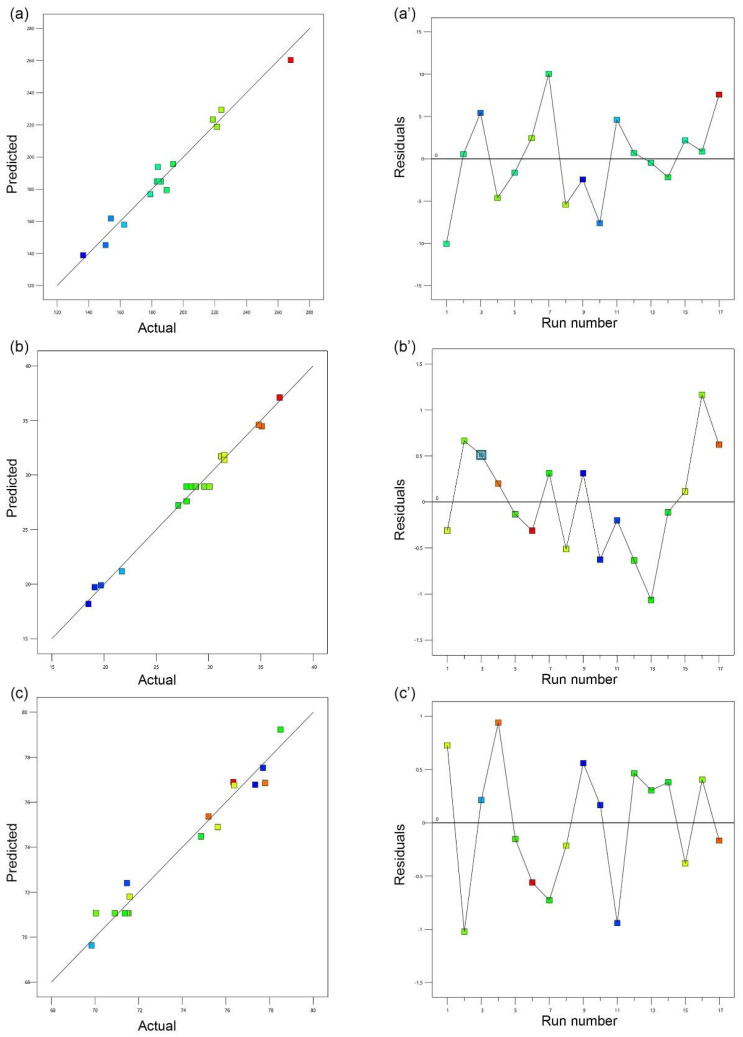
Liner correlation plot (**a**–**c**) between predicted and actual values and corresponding residual plot (**a’**–**c’**) for the three responses, i.e., mean particle size (nm), ZP (mV), and EE(%) for the CUR/LCSNPs. The scattered residual plot indicates the spread of the dependent variables under 17 different experimental conditions (run number).

The linear correlation plot (*A*, *B*, and *C*) between predicted and observed values are presented in Figure 3. The prediction error (%) is regarded as an appropriate method for determining errors that may occur during the experiment [21]. The prediction error (%) is close to zero, indicating that the obtained values are precise in comparison to the target value [21,35,36,37,38]. Furthermore, the prediction error (%) ensured that the obtained regression equation is valid [32,33].

### 3.2. SEM and XRD Analysis

**Figure 4:**
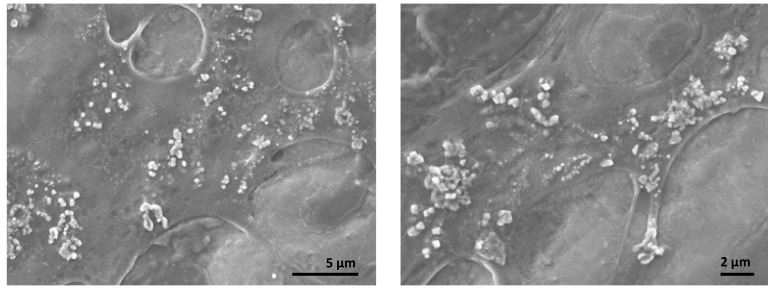
SEM images of CUR/LCSNPs at different magnifications.

**Figure 5:**
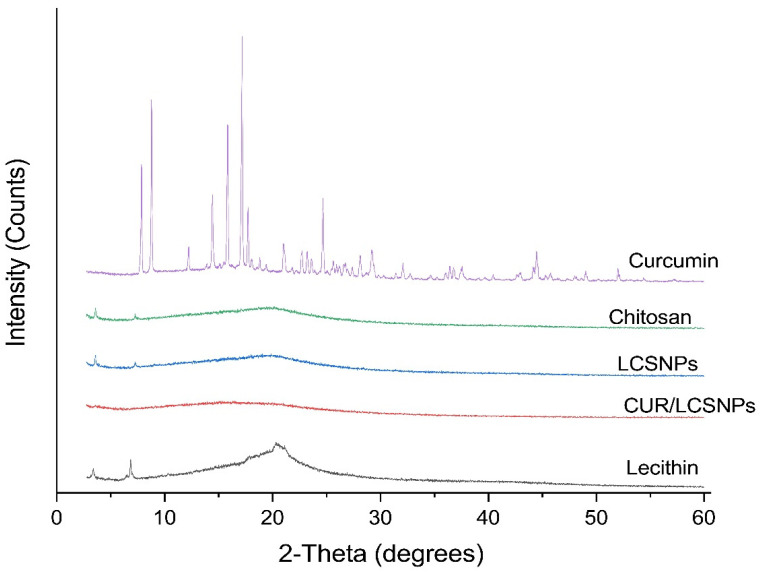
X-ray diffractograms of pure curcumin, lecithin, chitosan, LCSNPs, and CUR/LCSNPs.

Further analysis with SEM and XRD confirmed the presence of synthesized nanoparticles and the loading of curcumin onto LCSNPs. SEM images in Figure 4 show that CUR/LCSNPs have an irregular nanostructured shape, as well as aggregated nanoparticles. Comparative XRD spectra of pure curcumin, lecithin, chitosan, LCSNPs, and CUR/LCSNPs are presented in Figure 5. An XRD diffractogram of pure curcumin showed a series of intense peaks at 7.87°, 8.77°, 12.25°, 14.43°, 15.85°, 17.17°, 17.71°, 21.05°, 22.73°, 23.25°, 24.65°, 28.13°, and 29.23° at diffraction angles at the 2-theta axis, indicating the crystallinity nature of the curcumin as reported in the literature [39]. In the prepared CUR/LCSNPs, these peaks’ intensity was significantly reduced, indicating less crystallin (Figure 5). Thus, it indicated that the curcumin was adequately loaded within the chitosan in the nanoparticles. 

### 3.3. In Vitro Release Kinetics of CUR/LCSNPs

**Figure 6:**
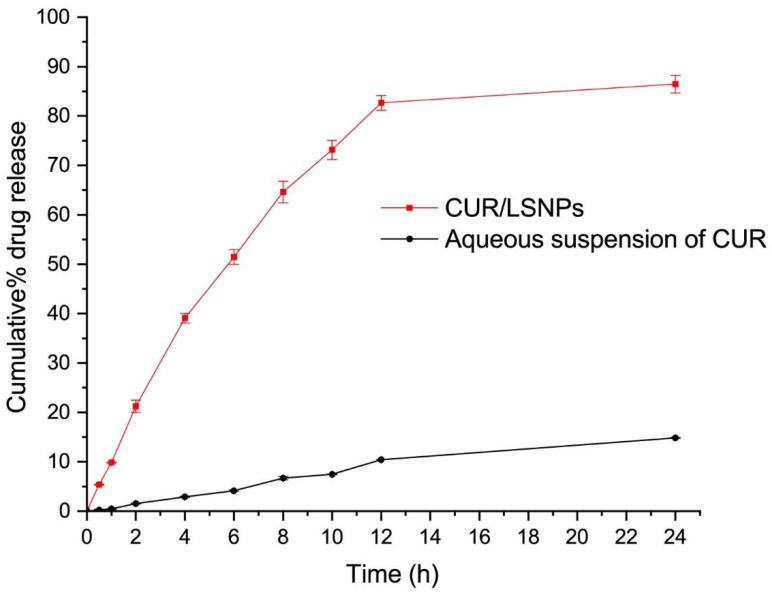
Cumulative percentage of the drug released from optimized CUR/LCSNPs in comparison to the aqueous suspension of the CUR.

To investigate the in vitro release profile of CUR for CUR/LCSNPs at pH 7.4, ethanol (5%) was used as a co-solubilizer to facilitate the release of CUR from the CUR/LCSNPs. The addition of 5% ethanol in the release media will not alter the release of CUR from CUR/LCSNPs, since it was previously investigated that ethanol concentration up to 40% *v*/*v* did not interfere with the release behavior of lipophilic drugs [40]. The in vitro release study demonstrated the slow and sustained release profile (Figure 6). The optimized CUR/LCSNPs formulation demonstrated 51.46 ± 1.49% of cumulative release of CUR in 6 h and 86.18 ± 1.5% of cumulative release of CUR in 24 h from the developed system at pH 7.4 (Figure 6). On the other hand, only 14.81 ± 0.10% of CUR was released from the aqueous suspension of CUR in 24 h at pH 7.4. The release of a drug from any formulation is affected by a variety of variables, including the dissolution and absorption of the drug from the aqueous media, the swelling behavior of the polymers, and the diffusion of the drug throughout the polymer network [41]. When exposed to aqueous media, CS hydrates to form a gelatinous layer, allowing the drug to be released from its matrix [42]. A major regulator of this process is the diffusion of the drug through the swollen matrix of the CS, as well as the erosion of the swollen matrix of the CS [43]. Various drug-release kinetic models were fit to the drug-release behavior of the optimized CUR/LCSNPs (Supplementary Figure S1). According to the overall curve fitting (Table 6), the release of CUR from the optimized CUR/LCSNPs followed the Korsmeyer–Peppas model. This indicates that the CUR release from the optimized CUR/LCSNPs is diffusion-controlled and follows Fickian diffusion (*n* < 0.45) [44]. 

### 3.4. Physical Stability Study

**Figure 7:**
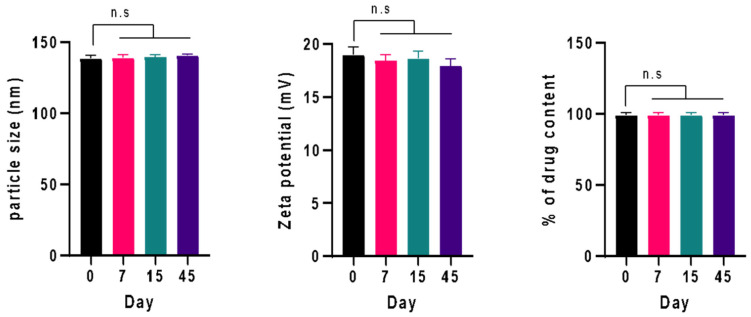
Physical stability of the optimized formulation of CUR/LCSNPs within 45 days. The difference among groups was analyzed using one-way ANOVA followed by Dunnet’s multiple comparisons to the mean value of day 0 (first column).

The physical stability of the optimized formulation of CUR/LCSNPs for 45 days was performed at 30 ± 1 °C. CUR/LCSNPs did not show any significant changes in the average particle size, PDI, zeta potential, and % drug content (Table 7 and Figure 7). At the end of 45 days, CUR/LCSNPs exhibited a slight increase in mean particle size due to CS’ swelling or adsorption to water [21]. 

## 4. Conclusions

Self-assembled CUR/LCSNPs with high %EE, appropriate drug release, and good physical stability at room temperature were developed by the application of a Box-Behnken design. CUR was successfully encapsulated into the matrix of LCSNPs. The quantitative effect of independent variables at various stages on achieving the smallest possible average particle size, the optimal zeta potential, and the highest possible %EE would be predicted using polynomial equations. The linear relationship between the predicted and actual values of the optimized formulation suggests that the response surface methodology is predictive. It was determined that the developed CUR/LCSNPs at a *w*/*w* ratio of 20:1 (L:CS) were suitable for further investigation in terms of average particle size, PDI, and zeta potential. The release kinetics of the optimized formulation suggested the release of CUR from the optimized CUR/LCSNPs formulation followed the Korsmeyer–Peppas model with Fickian diffusion (*n* < 0.45). We believe that the CUR-loaded LCSNPs developed in this study can serve as an optimal model for encapsulating hydrophobic drugs/bioactive for therapeutic/biomedical applications.

## Figures and Tables

**Table 1:** Independent and dependent variables in the optimization of CUR/LCSNPs through a Box-Behnken statistical design.

Factors	Level Used		
	Low (−1)	Medium (0)	High (+1)
Independent variables			
*A* = Chitosan (mg)	5	12.5	20
*B* = Lecithin (mg)	50	75	100
*C* = Speed (rpm)	900	1200	1500

Dependent variables: Y_1_ = Particle size (nm), Y_2_ = Zeta potential (mV), Y_3_ = Entrapment efficiency (%).

**Table 2:** CUR/LCSNPs experimental runs and observed responses for all of the seventeen formulations (F1–F17) exploiting the Box-Behnken statistical design.

	Independent Variables			Dependent Variables		
	*A* (mg)	*B* (mg)	*C* (rpm)	Y_1_ (nm)	Y_2_ (mV)	Y_3_ (%)
**F1**	12.5	50	900	183.74 ± 1.70	31.5 ± 1.25	75.62 ± 2.60
**F2**	12.5	75	1200	185.33 ± 3.50	29.6 ± 1.15	70.04 ± 2.40
**F3**	5	50	1200	150.63 ± 4.90	21.7 ± 2.10	69.84 ± 1.50
**F4**	20	75	1500	218.63 ± 2.70	34.8 ± 2.10	77.79 ± 2.90
**F5**	12.5	75	1200	183.15 ± 2.70	28.8 ± 1.40	70.91 ± 1.60
**F6**	20	50	1200	221.22 ± 4.20	36.8 ± 3.20	76.33 ± 2.10
**F7**	12.5	100	1500	189.40 ± 1.10	27.9 ± 2.20	78.50 ± 2.10
**F8**	20	100	1200	224.01 ± 4.30	31.2 ± 12.0	71.59 ± 2.50
**F9**	5	100	1200	136.44 ± 1.70	18.5 ± 1.40	77.34 ± 2.20
**F10**	5	75	1500	154.10 ± 2.10	19.1 ± 1.20	77.69 ± 2.70
**F11**	5	75	900	162.43 ± 2.50	19.7 ± 1.10	71.46 ± 2.30
**F12**	12.5	75	1200	185.48 ± 4.30	28.3 ± 2.40	71.53 ± 2.70
**F13**	12.5	75	1200	184.32 ± 3.10	27.9 ± 2.40	71.37 ± 3.0
**F14**	12.5	100	900	193.45 ± 3.50	27.1 ± 2.15	74.86 ± 2.50
**F15**	12.5	50	1500	179.01 ± 1.90	31.5 ± 1.90	76.37 ± 2.60
**F16**	12.5	75	1200	185.66 ± 3.10	30.1 ± 1.80	71.47 ± 2.30
**F17**	20	75	900	267.94 ± 3.70	35.1 ± 3.20	75.20 ± 2.90

**Table 3:** Best-fitted model for all three dependent responses (particle size, zeta potential, and entrapment efficiency of CUR/LCSNPs).

Response	Model	R-Squared (r^2^)	Adjusted r^2^	Predicted r^2^	Std. Dev.	%CV	Adequate Precision	Remarks
**Particle size (nm)**	Linear	0.9513	0.9401	0.9010	6.86	-	-	-
	Second order (2FI)	0.9206	0.8730	0.6111	11.19	-	-	-
	Quadratic	0.9984	0.9834	0.9389	4.57	2.44	47.4997	Suggested
**Zeta potential (mV)**	Linear	0.9481	0.9360	0.9028	1.41	-	-	-
	Second order (2FI)	0.9513	0.9220	0.7981	1.55	-	-	-
	Quadratic	0.9896	0.9762	0.9411	0.86	3.04	28.75	Suggested
**%EE**	Linear	0.8778	0.8229	0.6338	3.08	-	-	-
	Second order (2FI)	0.8643	0.8429	0.6580	2.83	-	-	-
	Quadratic	0.9837	0.9670	0.9378	0.88	1.19	14.20	Suggested

**Table 4:** ANOVA test for the dependent variables (particle size, zeta potential, and entrapment efficiency of CUR/LCSNPs).

Source	Sum of Square	Degree of Freedom	Mean Square	F value	*p*-Value	Remarks
**Y_1_ (nm)**						
Model-Quadratic	15,323.95	9	1702.66	26.81	0.0001	significant
*A*-Chitosan	13,464.08	1	13,464.08	211.99	<0.0001	
*B*-Lecithin	9.43	1	9.43	0.1485	0.7114	
*C*-Speed	551.62	1	551.62	8.69	0.0215	
*AB*	71.99	1	71.99	1.13	0.3224	
*AC*	419.86	1	419.86	6.61	0.0369	
*BC*	0.1146	1	0.1146	0.0018	0.9673	
*A* ^2^	168.61	1	168.61	2.65	0.1473	
*B* ^2^	272.47	1	272.47	4.29	0.0771	
*C* ^2^	392.63	1	392.63	6.18	0.0418	
Lack of fit	440.16	3	146.72	1.468	0.7217	Not significant
Pure error	4.43	4	1.11			
Total correlation	15,768.54	16				
**Y_2_ (mV)**						
Model-Quadratic	489.50	9	54.39	73.82	<0.0001	significant
*A*-Chitosan	433.65	1	433.65	588.56	<0.0001	
*B*-Lecithin	35.28	1	35.28	47.88	0.0002	
*C*-Speed	0.0012	1	0.0012	0.0017	0.9683	
*AB*	1.44	1	1.44	1.95	0.2048	
*AC*	0.0225	1	0.0225	0.0305	0.8662	
*BC*	0.1600	1	0.1600	0.2172	0.6554	
*A* ^2^	18.65	1	18.65	25.31	0.0015	
*B* ^2^	0.2047	1	0.2047	0.2778	0.6144	
*C* ^2^	0.5026	1	0.5026	0.6822	0.4361	
Lack of fit	1.80	3	0.6008	0.7163	0.5918	not significant
Pure error	3.36	4	0.8388			
Total correlation	494.66	16				
**Y_3_ (%EE)**						
Model-Quadratic	144.09	9	16.01	20.64	0.0003	significant
*A*-Chitosan	2.61	1	2.61	3.37	0.1090	
*B*-Lecithin	2.11	1	2.11	2.72	0.1429	
*C*-Speed	21.86	1	21.86	28.18	0.0011	
*AB*	37.45	1	37.45	48.30	0.0002	
*AC*	3.30	1	3.30	4.26	0.0780	
*BC*	2.09	1	2.09	2.69	0.1448	
*A* ^2^	3.85	1	3.85	4.96	0.0612	
*B* ^2^	13.00	1	13.00	16.76	0.0046	
*C* ^2^	52.13	1	52.13	67.22	<0.0001	
Lack of fit	3.89	3	1.30	3.37	0.1358	not significant
Pure error	1.54	4	0.3850			
Total correlation	149.52	16				

**Table 5:** Predicted and observed response of CUR/LCSNPs with maximum desirability suggested by the Box-Behnken statistical design.

Response	Predicted Value	Observed Value	Prediction Error (%) $=\frac{Observed-Predicted}{Predicted}\times 100$
**Particle size**	141.40	138.4 ± 2.10	−2.09%
**Zeta potential**	+18.19	+18.98 ± 0.72	4.36%
**%EE**	76.78	77.40 ± 1.70	0.79%

**Table 6:** Kinetic modeling of CUR release from the optimized CUR/LCSNPs.

Formulation	Zero-Order	First-Order	Higuchi Model	Hixon–Crowell	Korsmeyer–Peppas Model	
	R^2^	R^2^	R^2^	R^2^	R^2^	n
**Optimized CUR/LCSNPs**	0.7918	0.9794	0.9876	0.9747	0.9937	0.343
**Aqueous suspension of CUR**	0.8812	0.9786	0.9875	0.9833	0.9245	0.519

**Table 7:** Physical stability of the optimized formulation of CUR/LCSNPs within 45 days.

	Periods			
Parameter (Mean ± SD, n = 3)	0 Days	7 Days	15 Days	45 Days
**Particle size (nm)**	138.43 ± 2.09	138.97 ± 2.05	139.56 ± 1.55	140.54 ± 0.92
**PDI**	0.172 ± 0.031	0.175 ± 0.029	0.182 ± 0.024	0.188 ± 0.025
**Zeta potential (mV)**	+18.98 ± 0.72	18.46 ± 0.53	18.63 ± 0.68	17.93 ± 0.66
**%drug content**	99.19 ± 1.71	99.01 ± 1.77	98.97 ± 1.88	98.95 ± 1.92

## Data Availability

The data presented in this study are available in article or Supplementary Materials.

## Supplementary Materials

The following supporting information can be downloaded at: https://www.mdpi.com/article/10.3390/polym14183758/s1, Figure S1: Schematic illustration highlight various drug release kinetic models that were fitted to the drug release behavior of the optimized CUR/LCSNPs. High resolution figures is provided for Figure 1, Figure 2 and Figure 3.
