# Supplementary material for: Development of a Curcumin-Loaded Lecithin/Chitosan Nanoparticle Utilizing a Box-Behnken Design of Experiment: Formulation Design and Influence of Process Parameters
Source: Polymers (Basel). 2022 Sep 8;14(18):3758. doi: 10.3390/polym14183758 (PMC9505816; doi:10.3390/polym14183758)
Supplement: Supplementary file 1 [file polymers-14-03758-s001.zip › Supplimentary Figure S1.pdf]

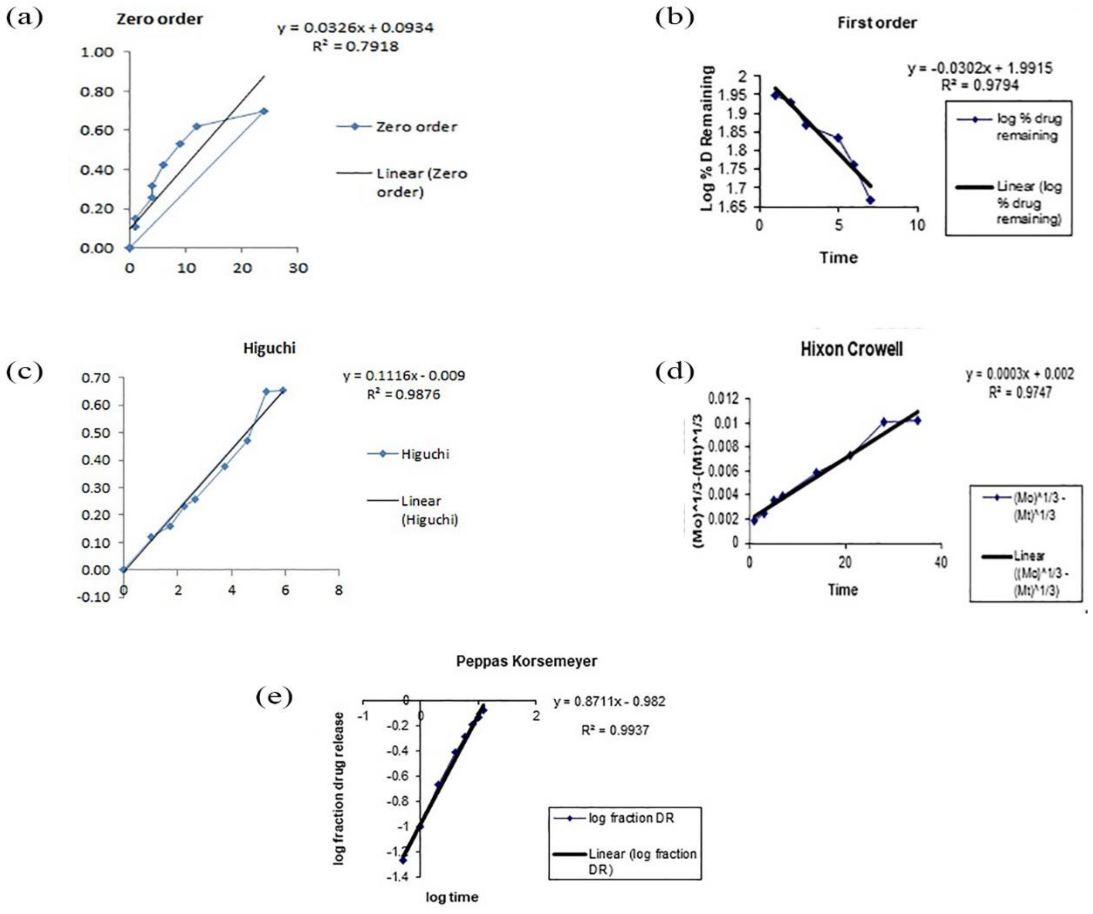

Figure S1. Schematic illustration highlight various drug release kinetic models that were fitted to the drug release behavior of the optimized CUR/LCSNPs.
